# Salinity reduction benefits European eel larvae: Insights at the morphological and molecular level

**DOI:** 10.1371/journal.pone.0198294

**Published:** 2018-06-13

**Authors:** Sebastian N. Politis, David Mazurais, Arianna Servili, Jose-Luis Zambonino-Infante, Joanna J. Miest, Jonna Tomkiewicz, Ian A. E. Butts

**Affiliations:** 1 National Institute of Aquatic Resources, Technical University of Denmark, DTU, Lyngby, Denmark; 2 Ifremer, Marine Environmental Science Laboratory UMR 6539, Plouzané, France; 3 Helmholtz Centre for Ocean Research, GEOMAR, Kiel, Germany; National Cheng Kung University, TAIWAN

## Abstract

European eel (*Anguilla anguilla*) is a euryhaline species, that has adapted to cope with both, hyper- and hypo-osmotic environments. This study investigates the effect of salinity, from a morphological and molecular point of view on European eel larvae reared from 0 to 12 days post hatch (dph). Offspring reared in 36 practical salinity units (psu; control), were compared with larvae reared in six scenarios, where salinity was decreased on 0 or 3 dph and in rates of 1, 2 or 4 psu/day, towards iso-osmotic conditions. Results showed that several genes relating to osmoregulation (*nkcc2α*, *nkcc2β*, *aqp1dup*, *aqpe*), stress response (*hsp70*, *hsp90*), and thyroid metabolism (*thrαA*, *thrαB*, *thrβB*, *dio1*, *dio2*, *dio3*) were differentially expressed throughout larval development, while *nkcc1α*, *nkcc2β*, *aqp3*, *aqp1dup*, *aqpe*, *hsp90*, *thrαA* and *dio3* showed lower expression in response to the salinity reduction. Moreover, larvae were able to keep energy metabolism related gene expression (*atp6*, *cox1*) at stable levels, irrespective of the salinity reduction. As such, when reducing salinity, an energy surplus associated to reduced osmoregulation demands and stress (lower *nkcc*, *aqp* and *hsp* expression), likely facilitated the observed increased survival, improved biometry and enhanced growth efficiency. Additionally, the salinity reduction decreased the amount of severe deformities such as spinal curvature and emaciation but also induced an edematous state of the larval heart, resulting in the most balanced mortality/deformity ratio when salinity was decreased on 3 dph and at 2 psu/day. However, the persistency of the pericardial edema and if or how it represents an obstacle in further larval development needs to be further clarified. In conclusion, this study clearly showed that salinity reduction regimes towards iso-osmotic conditions facilitated the European eel pre-leptocephalus development and revealed the existence of highly sensitive and regulated osmoregulation processes at such early life stage of this species.

## Introduction

Eels (*Anguilla spp*.) are euryhaline species that have adapted to cope with both, hyper- and hypo-osmotic environments, likely due to regular salinity changes in their habitats (i.e. estuaries) and/or migrations between freshwater and marine environments to complete their life cycle [1]. Adult eels undertake a downstream (catadromous) reproductive migration towards oceanic spawning areas, while eel offspring undertake an upstream migration towards estuarine or freshwater environments [1]. Unfortunately though, the natural populations and their reproductive potential have declined to a historical minimum, mainly due to climatic and anthropogenic pressures during the different phases of the eel life cycle [2]. The most exploited and negatively impacted populations today are the endangered American (*A*. *rostrata*) eel and Japanese (*A*. *japonica*) eel [3–4] as well as the critically endangered European (*A*. *anguilla*) eel [5].

In this context, actions have been taken to circumvent the eel decline, by minimizing fishery and pollution pressure through restocking and habitat restoration action plans [2]. Additionally, research is being conducted to advance knowledge on assisted reproduction and subsequent early life history (ELH) rearing conditions, towards a sustainable aquaculture. After extensive efforts, breeding protocols using assisted reproductive technologies were developed for the Japanese eel, leading to the first production of eel leptocephali larvae in captivity [6]. The Japanese eel achievements formed the basis for eel research, which led to the development of artificial reproductive protocols of the American [7] and European eel [8–10]. Subsequent work sought to identify optimal environmental rearing conditions throughout the ELH of eels, such as temperature [11–13], light [14], and salinity [15–16].

Regarding salinity, most fish species are hyper-osmotic in freshwater, where plasma osmolality is higher than the environment and hypo-osmotic in seawater, where plasma osmolality is lower than the environment [17]. Thus, in freshwater fish need to actively take up ions to counteract the diffusive ion loss and osmotic water gain, while in seawater they need to maintain osmotic balance through a desalting process to counteract osmotic water loss [18]. Eel offspring naturally occur in a hypo-osmotic environment in the ocean [19]. Interestingly though, it was shown that reducing salinity during ELH rearing, results in better growth and survival of Japanese eel larvae [15]. In some fish species, osmoregulatory organs such as gills or kidneys are not fully or not at all developed during ELH, thus embryos and early larvae control their ion balance *via* chloride cells, commonly located in the yolk sac area and the epithelia covering the larval body surface [20–21]. Those extra-branchial chloride cells (ionocytes) were also found to be located in the epithelium of the Japanese eel larval body surface and particularly abundant in the abdominal region, while forming multicellular complexes and influencing ionoregulation during ELH [22]. Furthermore, it was discovered that Japanese eel larvae can drink as early as the day of hatch, revealing that the role of gastro-intestinal osmoregulation starts earlier than previously anticipated [16, 23, 24]. The role and timing of the intestine and rectum in controlling ion balance was further confirmed by expression of osmoregulatory related genes such as Na^+^ K^+^ Cl^-^ (*nkcc*) and Na^+^ Cl^-^ (*ncc*) cotransporters in Japanese eel larvae [16].

Moreover, a recent study, using a transcriptomic (microarray) approach allowed the identification of a large number of genes involved with or affected by osmoregulatory changes during salinity adaptation in the European eel [25]. Furthermore, the European eel genome was recently sequenced and assembled [26], offering new perspectives for eel research in order to gain further knowledge regarding the molecular biology of this species. Thus, it is increasingly possible to follow targeted expression of genes involved in specific molecular mechanisms such as sodium potassium chloride ion cotransporters, which mediate the electroneutral cotransport of Na^+^, K^+^ and Cl^-^ and are known to be involved in ion absorption and/or secretion as well as in cell volume homeostasis [27]. An additional mechanism of interest involves aquaporins, which are membrane proteins, forming pores to selectively facilitate rapid transport and exchange of water molecules in addition to diffusion through the plasma membrane [28]. Also an important mechanism controlling cellular homeostasis, is the molecular response to extrinsic (environmental) stressors. Here, heat shock proteins which are produced in all cellular organisms, regulate normal protein synthesis, and play an important role in an organism’s health as they are expressed in response to stress in order to counteract physiological injury and reduce trauma [29]. A different molecular mechanism of interest is the thyroid endocrine system, as it has been shown to be involved in various physiological processes and contributes to homeostasis regulation [30]. Furthermore, the oxidative phosphorylation (OXPHOS) pathway represents a mechanism of importance, where genes associated to energy metabolism are today well known in teleost fishes [31].

In this context, this study investigates how salinity affects European eel larval biometry (such as morphology and growth), deformities and survival as well as expression profiles of genes related to processes involved in or affected by osmoregulation, such as ion transport [Na^+^K^+^2Cl^-^ cotransporters (*nkcc*), aquaporins (*aqp*)], energy metabolism [mitochondrial ATP Synthase F0 subunit 6 (*atp6*), cytochrome C oxidase 1 (*cox1*)], thyroid metabolism [thyroid hormone receptors (*thr*), deiodinases (*dio*)] and stress response [heat shock proteins (*hsp*)]. We hypothesized that lower salinity towards a more iso-osmotic environment, already at a very early stage, would reduce stress and conserve energy due to lower cost for osmoregulation, resulting in higher survival and growth during the ELH stages but also facilitating a greater larval developmental potential. Thus, the main objective of this study was to determine the optimal condition for rearing European eel pre-leptocephalus larvae by decreasing salinity on 0 or 3 days post hatch (dph) and at rates of 1, 2 or 4 psu/day.

## Material and methods

### Ethics statement

All fish were handled in accordance with the European Union regulations concerning the protection of experimental animals (Dir 86/609/EEC). Eel experimental protocols were approved by the Animal Experiments Inspectorate (AEI), Danish Ministry of Food, Agriculture and Fisheries (permit number: 2015-15-0201-00696). Briefly, adult eels were anesthetized using ethyl p-aminobenzoate (benzocaine) before tagging and handling. Endogenously feeding larvae of European eel were anesthetized prior to handling and euthanized prior to sampling by using tricaine methanesulfonate (MS-222). All efforts were made to minimize animal handling and stress.

### Broodstock management

Female broodstock were wild-caught from lake Vandet (Denmark) or Lough Neagh (Ireland), while all males originated from a commercial eel farm (Stensgård Eel Farm A/S, Denmark). After collection, broodstock were transferred to an experimental facility of the Technical University of Denmark, where they were maintained in ~1250 L polyethylene tanks equipped with a closed recirculation system, under a continuous flow rate of ~10–15 L min^−1^, low intensity light (~20 lux) and 12 h day/12 h night photoperiod. Acclimatization took place over two weeks, in order to reach a salinity of 36 psu and temperature of 20°C. As eels naturally undergo a fasting period from the onset of the pre-pubertal silvering stage, they were not fed during this period. Prior to experimentation, the broodstock were anaesthetized (ethyl p-aminobenzoate, 20 mg L^−1^; Sigma-Aldrich Chemie, Steinheim, Germany), tagged with a passive integrated transponder, and length and weight recorded.

### Gamete production, experimental design and conditions

To induce vitellogenesis females received weekly injections of salmon pituitary extract at 18.75 mg kg^−1^ body weight (Argent Chemical Laboratories, Washington, USA) [10, 32]. To stimulate follicular maturation and induce ovulation, females received an additional injection of 17α,20ß-dihydroxy-4-pregnen-3-one (Sigma-Aldrich, St. Louis, MO, USA) at 2.0 mg kg^−1^ body weight [33]. Then, within 12–14 h, eggs were stripped from females. Males received weekly injections of human chorionic gonadotropin (hCG, Sigma Aldrich Chemie, Steinheim, Germany) at 150 IU/fish. Prior to fertilization, they were given another injection and milt was collected ~12 h after administration of hormone. Milt samples were pipetted into an immobilizing medium [34] and used for fertilization within 4 h of collection [35].

The experiment was repeated 3 times, each time using a different experimental cross. Eggs from each female were “crossed” with a sperm pool of several males to experimentally create the 3 experimental crosses. Eggs from each female were stripped into dry plastic containers and gametes were swirled together. Artificial seawater (20°C and 36 psu) prepared by using reverse osmosis filtration (Vertex Puratek 100 gpd RO/DI, Vertex Technologies Inc., CA, USA) and salted with Red Sea Salt (Red Sea, Red Sea International, Eilat, Israel) was added for a gamete contact time of 5 min [35, 36]. Eggs/embryos were then incubated in 15 L of the above described artificial seawater (18°C and 36 psu) [13] and supplemented with rifampicin and ampicillin (each 50 mg L^-1^, Sigma-Aldrich, Missouri, USA) until hatch [37].

At hatch, larvae were randomly distributed (~1000 individuals per replicate) into 1 L glass beakers and reared throughout the endogenous feeding stage, from 0 to 12 dph, with no aeration [38]. Each beaker was filled with 800 mL of artificial seawater (18°C and 36 psu) and supplemented with antibiotics as previously described [13, 37]. Using a factorial approach, salinity treatments were step-wise adjusted beginning on 0 or 3 dph and at rates of 1, 2, or 4 psu/day, in order to reach a more iso-osmotic condition compared to full strength seawater (control), which was kept at 36 psu. This resulted in overall seven salinity treatments (con, 01, 02, 04, 31, 32 and 34) described in Table 1. All 3 experimental crosses where represented in 3 replicates for all 7 treatments, resulting in 63 experimental beakers. Each day, 400 mL were removed and replaced with pre-adjusted artificial seawater, in order to reach the desired salinity of each treatment, which was measured using a digital portable conductivity meter (WTW ProfiLine Cond 3110). All treatments underwent the same handling procedures. Rearing of embryos and larvae took place in darkness, while handling and sampling under low intensity (< 2.2 μmol m^-2^ s^-1^) light conditions [14].

**Table 1:** European eel (*Anguilla anguilla*) larvae reared in seven different salinity treatments; at 36 psu (con) and in six further scenarios, where salinity was reduced on 0 or 3 days post hatch and at rates of 1, 2 or 4 psu/day (01, 02, 04, 31, 32 and 34) towards iso-osmotic conditions.

			Age in days post hatch (dph)												
Reduction		ID	0	1	2	3	4	5	6	7	8	9	10	11	12
dph	psu day^-1^		Salinity (psu)												
Control		con	36												
0	1	**01**	35	34	33	32	31	30	29	28	27	26	25	24	23
	2	**02**	34	32	30	28	26	24	22	20	18	16			
	4	**04**	32	28	24	20	16								
3	1	**31**	36			35	34	33	32	31	30	29	28	27	26
	2	**32**	36			34	32	30	28	26	24	22	20	18	16
	4	**34**	36			32	28	24	20	16					

### Data collection

Larval development (biometry), mortality, and gene expression of selected genes, corresponding to specific molecular mechanisms were followed from hatch and throughout the endogenous feeding stage (2, 4, 6, 8, 10, and 12 dph). All endogenously feeding larvae of European eel were anesthetized using tricaine methanesulfonate (MS-222; Sigma-Aldrich, Missouri, USA) prior to digital imaging and euthanized post-sampling by using an MS-222 overdose [13]. All images were taken using a digital camera (Digital Sight DS-Fi2, Nikon Corporation, Japan) attached to a zoom stereomicroscope (SMZ1270i, Nikon Corporation, Japan), while NIS-Elements D analysis software (Version 3.2) was used to analyze the larval images (Nikon Corporation, Japan).

#### Mortality and biometry

Every day, dead larvae were counted and removed from all experimental units. Additionally, all larvae at the end of the experiment as well as of all sampled larvae from each experimental unit were enumerated and recorded. Larval cumulative mortality was calculated as a percentage from hatch until 12 dph.

**Fig 1:**
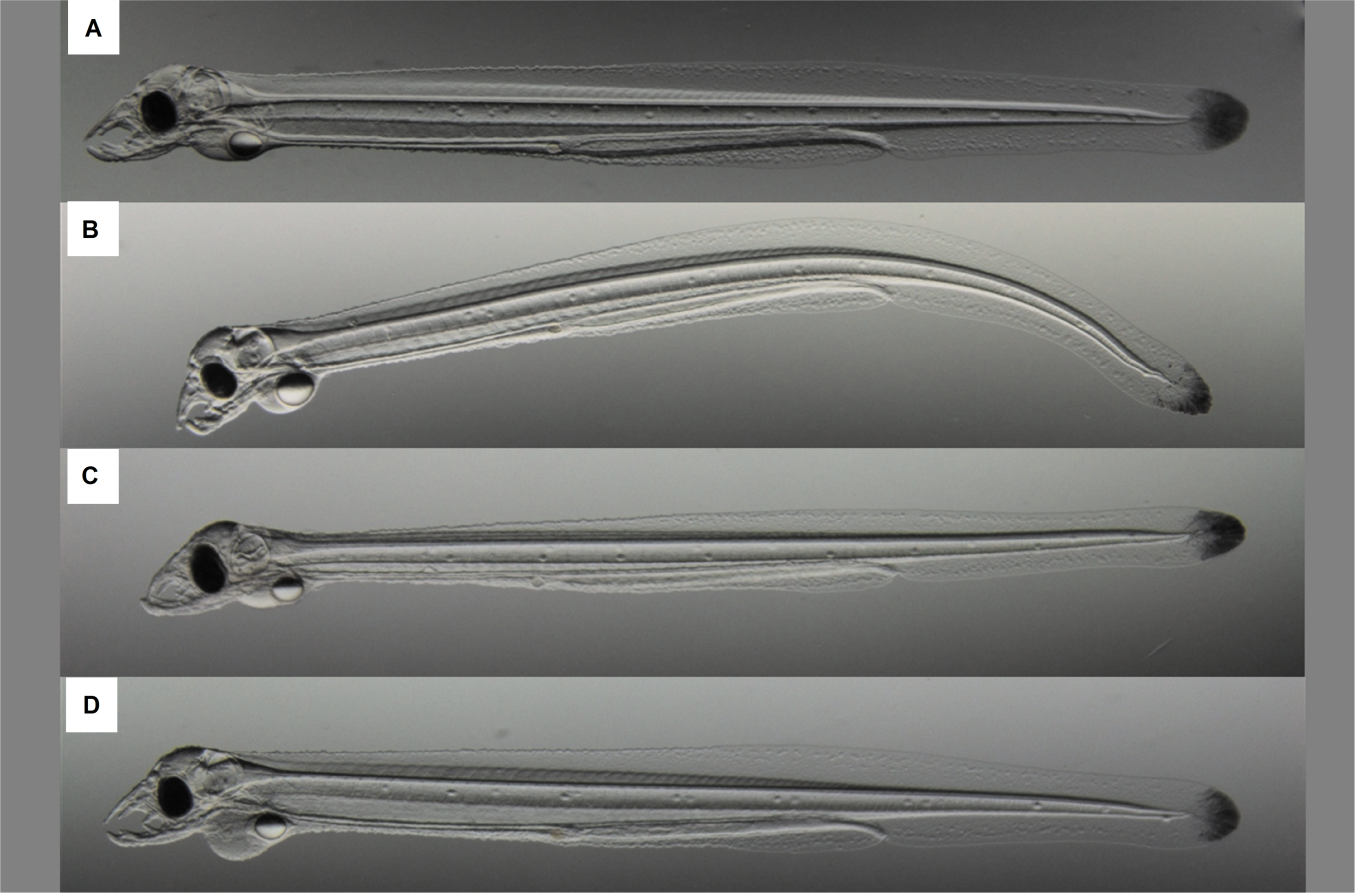
Visualization of European eel (*Anguilla anguilla*) larval deformities. Normal (A), spinal curvature (B), emaciation (C), and pericardial edema (D).

For analysis of larval biometry, ~10 larvae from each replicate (3×), cross, and treatment were randomly sampled at hatch and every second day until 12 dph. Larvae were digitally imaged (as described above) for later analyses, where total body and oil-drop area were measured for each larva. Larval growth and oil-drop utilization were measured from the change in body and oil-drop area, respectively. Growth efficiency was measured by dividing the increase in body area by the corresponding decrease in oil-drop area. Moreover, larval deformities were classified according to [39] as spinal curvature, emaciation and pericardial edema (Fig 1).

#### Gene expression

For molecular analysis, ~30 larvae from each replicate, experimental cross, and treatment were randomly sampled at hatch and every second dph until the first-feeding stage. Those larvae were recorded, euthanized using MS-222, rinsed with deionized water, preserved in RNAlater Stabilization Reagent and kept at -20°C following the procedure suggested by the supplier (Qiagen, Hilden, Germany). RNA was extracted using the NucleoSpin^®^ RNA Kit (Macherey-Nagel, Germany) following the manufacturer’s instructions. RNA concentration (264 ± 230 ng μl^-1^) and purity (260/280 = 2.13 ± 0.03, 260/230 = 2.23 ± 0.12) were determined by spectrophotometry using Nanodrop ND-1000 (Peqlab, Germany) and normalized to a common concentration of 100 ng μl^-1^ with HPLC water. From the resulting total RNA, 680 ng were transcribed using the qScript^TM^ cDNA Synthesis Kit (Quantabio, Germany) according to the manufacturer’s instructions, including an additional gDNA wipe out step prior to transcription [PerfeCta^®^ DNase I Kit (Quantabio, Germany)].

The *ef1α* and rps*18* genes were chosen as housekeeping genes, since qBase+ software revealed that these mRNA levels were stable throughout analyzed samples (M < 0.4); M gives the gene stability and M < 0.5 is typical for stably expressed reference genes [40]. The expression levels of 16 target and 2 reference (*ef1α*, rps*18*) genes were determined by quantitative real-time PCR (RT-qPCR), using specific primers (Table 2). Primers were designed using primer 3 software v 0.4.0 (http://frodo.wi.mit.edu/primer3/) based on cDNA sequences available in GenBank Nucleotide, the European eel transcriptome database (EeelBase 2.0, http://compgen.bio.unipd.it/eeelbase/) or the available European eel genome (https://www.ncbi.nlm.nih.gov/bioproject/PRJNA73577). All primers were designed for an amplification size ranging from 75 to 200 nucleotides and optimal Tm of 60°C.

**Table 2:** Sequences of European eel, *Anguilla anguilla* primers used for amplification of genes by qRT-PCR. Primers were designed based on sequences available on Genbank databases. The table lists accession number and corresponding database of target gene sequences.

Full name	Abbreviation	Function	Database	Accession Number	Primer sequence (5’ 3’) (F: Forward; R: Reverse)
Na^+^K^+^2Cl^-^ Cotransporter 1α	*nkcc1α*	Ion transport	GenBank Nucleotide	AJ486858	F: CCAAGGCTCAGATCTTCCTG
					R: TTTCCGAATGGTAACCGAAG
Na^+^K^+^2Cl^-^ Cotransporter 2α	*nkcc2α*	Ion transport	GenBank Nucleotide	AJ564602	F: ACGTGGTTGGGTTTTCAGAG
					R: GTGAGATCCCCAAAAGCAAA
Na^+^K^+^2Cl^-^ Cotransporter 2β	*nkcc2β*	Ion transport	GenBank Nucleotide	AJ564603	F: AGCCAAAGTGGTGGATGTTC
					R: TGTCAGCCTCTCCAGTTCCT
Aquaporin 3	*aqp3*	Water transport	GenBank Nucleotide	AJ319533	F: GCTCTCATGGCTTGTTCCTC
					R: AAGGTCACAGTGGGGTTCAG
Aquaporin 1 duplicate	*aqp1dup*	Water transport	GenBank Nucleotide	AJ564421	F: GAATTCCTGGCAACCTTTCA
					R: CAAGATGACCCAGACCCACT
Aquaporin e	*aqpe*	Water transport	GenBank Nucleotide	AJ784153	F: TGGGCAGCTGACAGTAACAG
					R: AATCACCTGGTCCACAAAGC
Heat Shock Protein 70	*hsp70*	Stress response	GenBank WGS	AZBK01685255	F: TCAACCCAGATGAAGCAGTG
					R: GCAGCAGATCCTGAACATTG
Heat Shock Protein 90	*hsp90*	Stress response	GenBank WGS	AZBK01838994	F: ACCATTGCCAAGTCAGGAAC
					R: ACTGCTCATCGTCATTGTGC
ATP Synthase F0 subunit 6	*atp6*	Energy metabolism	GenBank Nucleotide	NC_006531	F: GGCCTGCTCCCATACACATT
					R: GACTGGTGTTCCTTCTGGCA
Cytochrome C Oxidase 1	*cox1*	Energy metabolism	GenBank Nucleotide	NC_006531	F: CTACTCCTCTCCCTGCCAGT
					R: CTTCTGGGTGGCCGAAGAAT
Deiodinase 1	*dio1*	Thyroid metabolism	EeelBase 2.0	Eeel2-c186	F: AGCTTTGCCAGAACGACTGT
					R: TTCCAGAACTCTTCGCACCT
Deiodinase 2	*dio2*	Thyroid metabolism	Eel genome website	g12347	F: GAAGAGGAGGATCGCCTACC
					R: GCACTCTACCTCCGTCCAAA
Deiodinase 3	*dio3*	Thyroid metabolism	EeelBase 2.0	Eeel-c22164	F: TACGGGGCGTATTTTGAGAG
					R: GCTATAACCCTCCGGACCTC
Thyroid Hormone Receptor α A	*thrαA*	Thyroid metabolism	GenBank Nucleotide	KY082904	F: GCAGTTCAACCTGGACGACT
					R: CCTGGCACTTCTCGATCTTC
Thyroid Hormone Receptor α B	*thrαB*	Thyroid metabolism	GenBank Nucleotide	KY082905	F: GAAGCCTTCAGCGAGTTCAC
					R: ACAGCCTTTCAGGAGGATGA
Thyroid Hormone Receptor β B	*thrβB*	Thyroid metabolism	GenBank Nucleotide	KY082907	F: GAAGACTGAGCCCTGAGGTG
					R: AGGTAATGCAGCGGTAATGG
Elongation Factor 1 α	*ef1a*	Housekeeping	GenBank Nucleotide	EU407824	F: CTGAAGCCTGGTATGGTGGT
					R: CATGGTGCATTTCCACAGAC
40S Ribosomal S18	*rps18*	Housekeeping	GenBank TSA	GBXM01005349	F: TGACCGATGATGAGGTTGAG
					R: GTTTGTTGTCCAGACCGTTG

Expression of genes in each larval sample from 2 randomly selected replicates, from each parental cross, treatment, and larval age (2, 4, 6, 8, 10 and 12 dph) were analysed in two technical replicates of each gene using the qPCR Biomark^TM^ HD system (Fluidigm) based on 96.96 dynamic arrays (GE chips) as previously described [41]. In brief, a pre-amplification step was performed with a 500 nM primer pool of all primers in TaqMan-PreAmp Master Mix (Applied Biosystems) and 1.3 μL cDNA per sample for 10 min at 95°C and then 14 cycles of 15 s at 95°C and 4 min at 60°C. Obtained PCR products were diluted 1:10 with low EDTA-TE buffer. The pre-amplified product was loaded onto the chip with SSofast-EvaGreen Supermix low Rox (Bio Rad) and DNA-Binding Dye Sample Loading Reagent (Fluidigm). Primers were loaded onto the chip at a concentration of 50 μM. The chip was run according to the Fluidigm 96.96 PCR protocol with a Tm of 60°C. The relative quantity of target gene transcripts was normalized and measured using the ΔΔ Ct method [42]. Coefficient of variation (CV) of technical replicates was calculated and checked to be < 0.04 [40].

### Statistical analyses

All data were analyzed using SAS statistical software (version 9.1; SAS Institute Inc., Cary, North Carolina). Residuals were tested for normality using the Shapiro-Wilk test and homogeneity of variances was tested using a plot of residuals versus fit values (PROC GLOT, SAS Institute 2003). Data were log_10_ or arcsine square-root-transformed when data deviated from normality or homoscedasticity [43]. The effect of salinity on larval deformities, growth rate, oil-drop utilization, and growth efficiency on 12 dph was determined using a series of one-way ANOVA models, where experimental cross was considered a random factor (SAS PROC MIXED; SAS Institute 2003). Tukey’s post-hoc analyses were used to compare least-squares means between treatments. Furthermore, statistical models were used to investigate salinity effects on larval body area, mortality and gene expression throughout early larval development (from 2 to 12 dph). Here, we analyzed the data using a series of repeated measures mixed-model ANOVAs (PROC MIXED; SAS Institute 2003). Models contained salinity treatment and age main effects as well as the treatment × age interaction. Akaike’s (AIC) and Bayesian (BIC) information criteria were used to assess which covariance structure (compound symmetry, autoregressive order, or unstructured) was most appropriate [44]. Salinity treatment and age were considered fixed, whereas parental cross was considered random. Tukey’s post-hoc analyses were used to compare least-squares means between treatments. If a significant salinity × age interaction was detected, the model was decomposed into a series of reduced ANOVA models to determine the effect of salinity for each age. This was the case only for *thrαA*.

## Results

### Mortality and biometry

**Fig 2:**
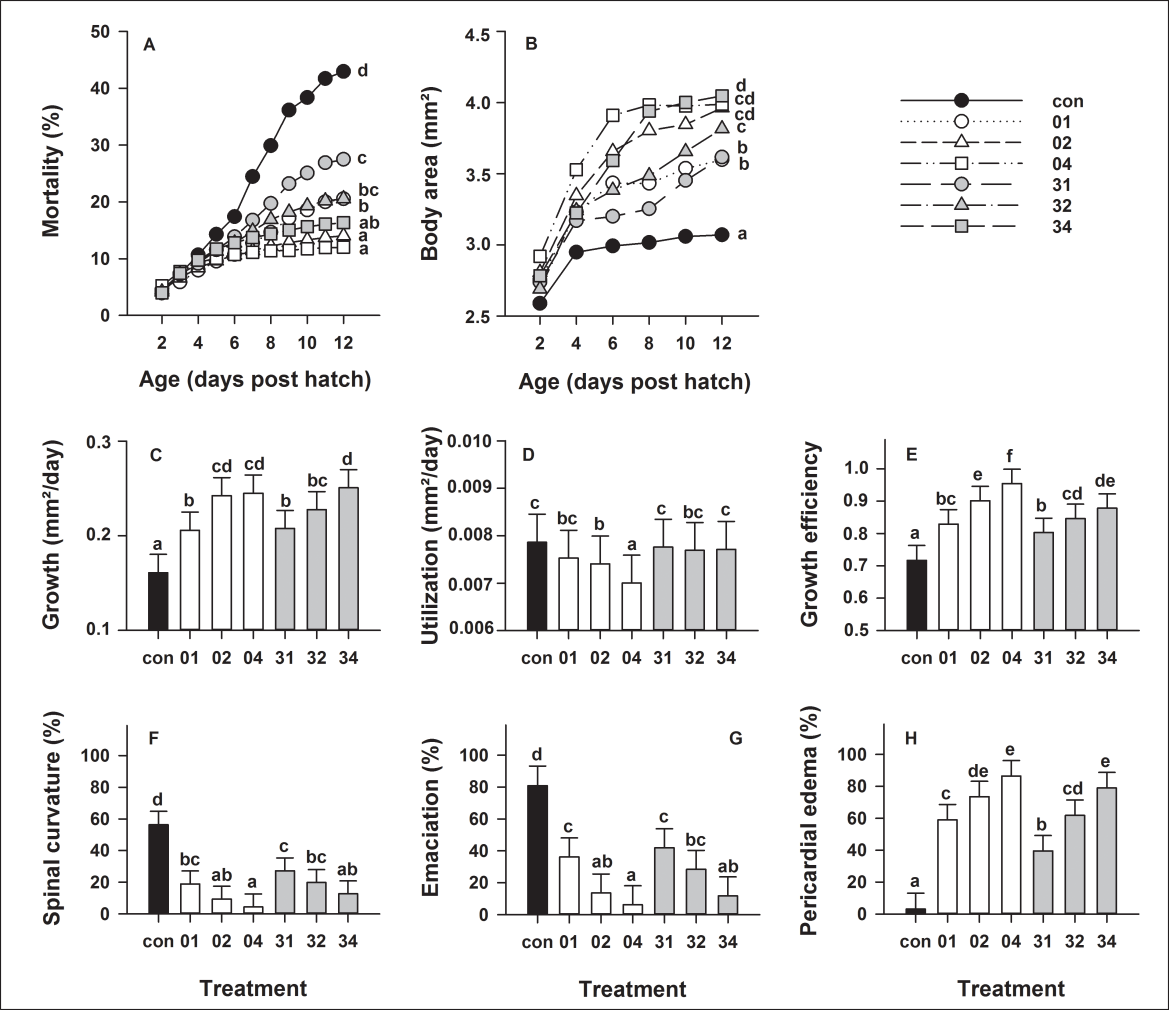
Effect of salinity on European eel (*Anguilla anguilla*) larval mortality and biometry. Mortality (A), body area (B), growth (C), oil drop utilization (D) and growth efficiency (E) from 2 to 12 days post hatch (dph) as well as occurrence of larval deformities such as spinal curvature (F), emaciation (G) and pericardial edema (H) on 12 dph. Values represent means (± SEM) among three crosses at each age and treatment. Different lower case letters represent significant statistical differences (p < 0.05).

European eel larval mortality (± SEM) until 12 dph, was highest (43 ± 10%) when larvae were reared at 36 psu (control) and significantly (p < 0.0001) lower in all salinity reduced treatments (Fig 2A). The statistically lowest larval mortality was observed when salinity was initially reduced on 0 dph and at 2 or 4 psu/day (treatments 02 and 04, respectively) as well as on 3 dph and at 4 psu/day (treatment 34).

Larval body area (±SEM), which increased until 12 dph, was found to be lowest (3.1 ± 0.2 mm^2^) when larvae were reared at 36 psu (control) and significantly (p < 0.0001) higher in all salinity reduced treatments (Fig 2B). Larvae developed the highest body area (3.8–4.0 ± 0.2 mm^2^) when salinity was initially reduced on 0 dph and at 2 or 4 psu/day (treatments 02 and 04, respectively) as well as on 3 dph and at 4 psu/day (treatment 34).

Larval growth (± SEM) throughout the endogenous feeding window was significantly (p < 0.0001) lower (0.16 ± 0.02 mm^2^ per day) when larvae were reared at 36 psu (control) compared to all other treatments (Fig 2C). Highest growth (> 0.24 ± 0.02 mm^2^/day) occurred when salinity was reduced on 0 dph and at 2 or 4 psu/day (treatments 02 and 04, respectively), as well as on 3 dph and at 4 psu/day (treatment 34). Oil drop utilization (± SEM) was significantly (p < 0.0001) lower (0.007 ± 0.0006 mm^2^/day) when salinity was reduced on 0 dph and at 4 psu/day (treatment 04) compared to all other treatments (Fig 2D). All salinity reduction treatments resulted in a growth efficiency (± SEM) that was significantly (p < 0.0001) higher compared to larvae reared at 36 psu (control; 0.72 ± 0.04), while highest growth efficiency (0.95 ± 0.04) occurred when salinity was reduced on 0 dph and at 4 psu/day (treatment 04; Fig 2E).

On 12 dph, the occurrence of larvae with spinal curvature (± SEM) were significantly (p < 0.0001) higher (56 ± 8%) when larvae were reared at 36 psu (control), compared to all salinity reduction treatments (Fig 2F), while lowest (4 ± 8%) occurrence of larvae with spinal curvature were detected when salinity was reduced on 0 dph and at 4 psu/day (treatment 04). Similarly, the occurrence of larvae with emaciation (± SEM) were significantly (p < 0.0001) higher (81 ± 12%) when larvae were reared at 36 psu (control), compared to all salinity reduction treatments (Fig 2G), while lowest (6 ± 8%) occurrence of larvae with emaciation were detected when salinity was reduced on 0 dph and at 4 psu/day (treatment 04). On the contrary, when salinity was reduced on 0 dph and at 2 or 4 psu/day (treatments 02 and 04, respectively), as well as on 3 dph and at 4 psu/day (treatment 34), we observed larvae with a significantly (p < 0.0001) higher (> 73 ± 10%) number of pericardial edema compared to larvae reared at 36 psu (control; Fig 2H).

### Gene expression

#### Osmoregulation

**Fig 3:**
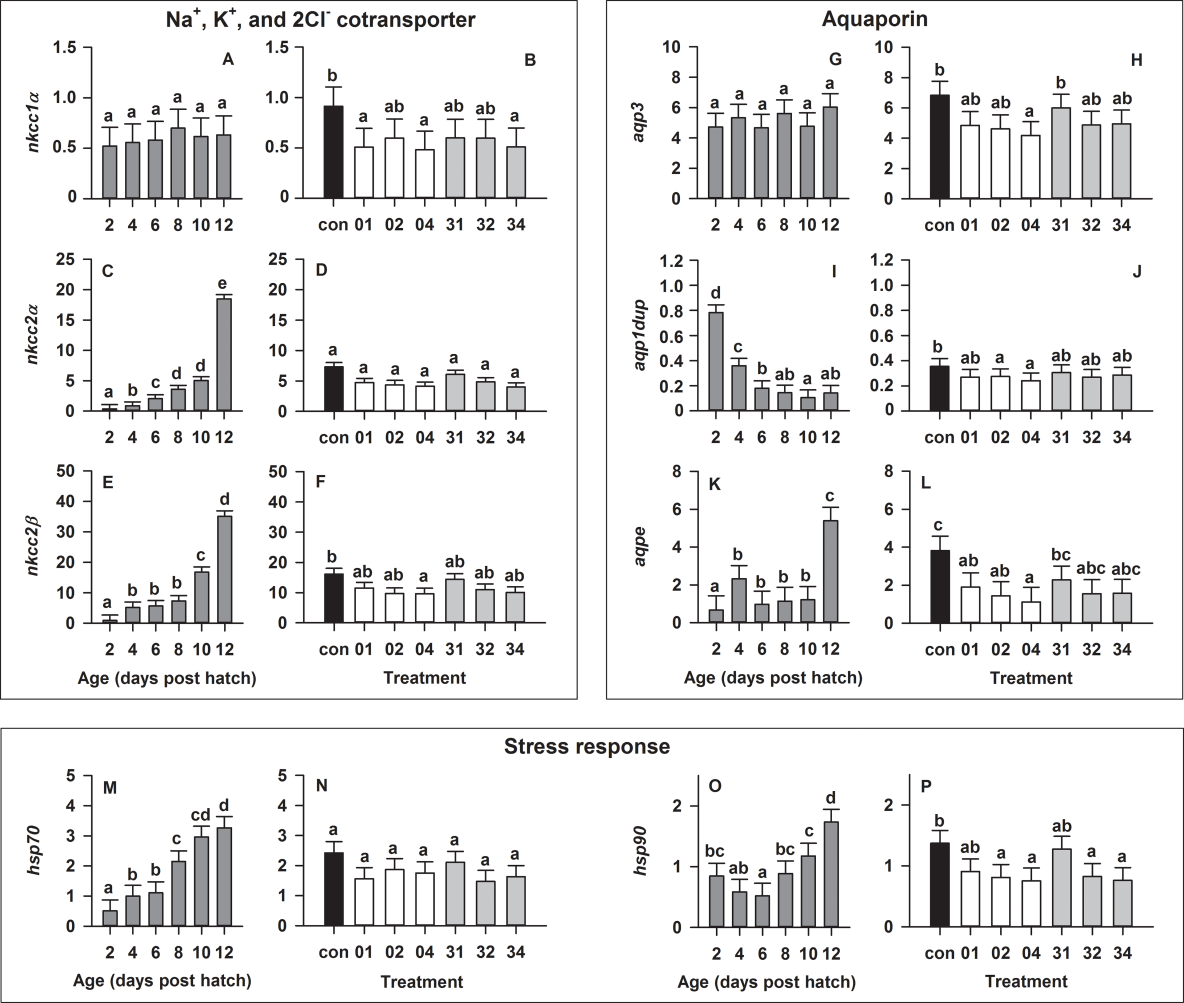
Effect of age and salinity treatment on European eel (*Anguilla anguilla*) larval relative expression of selected genes. No significant salinity × age interaction was detected for any gene. As such, over the entire experimental period, the main effects of age and salinity are displayed for genes encoding Na^+^K^+^2Cl^-^ cotransporters (A-F), aquaporins (G-L), and heat shock proteins (M-P). Values represent means (± SEM) among three crosses at each age and treatment. Different lower case letters represent significant statistical differences (p < 0.05).

Na^+^ K^+^ 2Cl^-^ cotransporter regulation were affected by both larval age and salinity. Relative expression levels of the genes encoding for *nkcc1α* were stable (Fig 3A), while *nkcc2α* and *nkcc2β* increased significantly (p < 0.0001) throughout larval development and peaked at 12 dph (Fig 3C and 3E). Expression of *nkcc1α* was significantly (p < 0.01) reduced when salinity was decreased on 0 dph and at 1 or 4 psu/day (treatments 01 and 04, respectively) as well as on 3 dph and at 4 psu/day (treatment 34; Fig 3B) compared to the 36 psu control. Similarly, expression of *nkcc2β* was significantly (p < 0.01) higher in the control group than when salinity was decreased on 0 dph and 4 psu/day (treatment 04; Fig 3F), while no statistically significant effect of salinity was observed on expression levels of *nkcc2α* (Fig 3D).

Moreover, the relative expression of aquaporin 3 (*aqp3*) was stable (Fig 3G), *aqp1dup* significantly (p < 0.0001) decreased (Fig 3I), while *aqpe* significantly (p < 0.0001) increased throughout larval development (Fig 3K). Expression of *aqp3* was significantly (p = 0.005) reduced when salinity was decreased on 0 dph and at 4 psu/day (treatment 04; Fig 3H) compared to no reduction (control) or the slowest reduction in treatment 31 (reduction on 3 dph and at 1 psu/day). Expression of *aqp1dup* was also significantly (p < 0.002) higher when larvae were reared at 36 psu (control) compared to when salinity was decreased on 0 dph and at 2 or 4 psu/day (treatments 02 and 04, respectively; Fig 3J). Similarly, the salinity reduction on 0 dph, irrespective of the reduction rate (treatments 01, 02 and 04; Fig 3L), caused a significant (p < 0.001) reduction in mRNA levels of the third aquaporin tested (*aqpe*).

#### Stress response

The relative expression of heat shock protein 70 *(hsp70)* significantly (p < 0.0001) increased throughout development, while *hsp90* experienced a significant (p < 0.0001) decrease from 2 to 6 dph, but increased again beyond that; both reaching a peak on 12 dph (Fig 3M and 3O). The rate of reduction was the driver setting the expression pattern for *hsp90*, since reduction rates of 2 and 4 psu/day let to lowered mRNA levels independent of the onset of the treatment (p < 0.001, Fig 3P). The expression response of *hsp70* showed a similar pattern to *hsp90*; however it was not statistically different among the treatments (Fig 3N).

#### Thyroid metabolism

**Fig 4:**
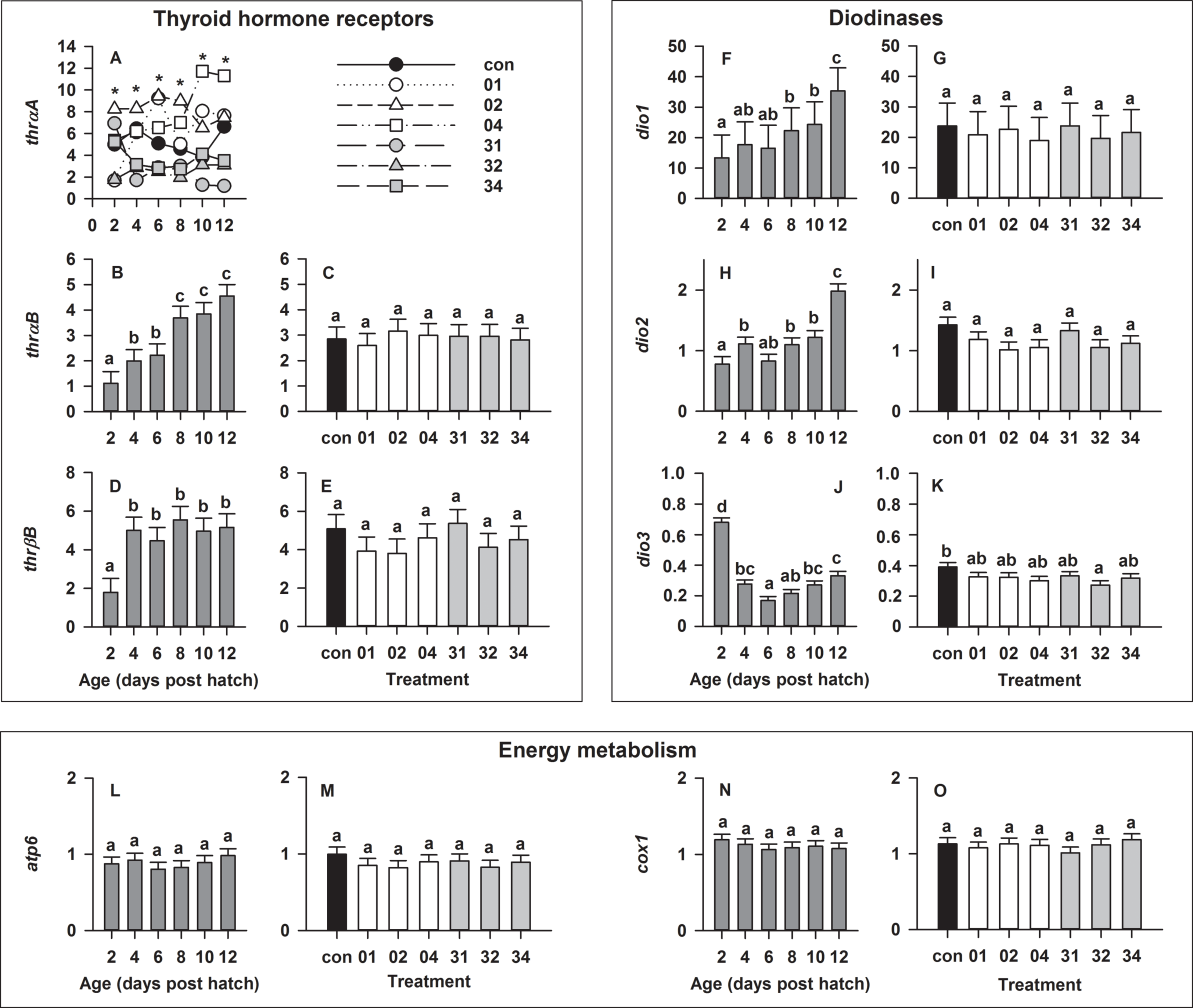
Effect of age and salinity treatment on European eel (*Anguilla anguilla*) larval relative expression of selected genes. For thyroid hormone receptor αA (*thrαA*), a significant salinity × age interaction was detected, thus the model was decomposed into a series of reduced ANOVA models to determine the effect of salinity for each age (A). No significant salinity × age interactions were detected for all other genes. As such, over the entire experimental period, the main effects of age and salinity are displayed for genes relating to thyroid hormone receptors (B-E), deiodinases (F-K), and energy metabolism (L-O). Values represent means (± SEM) among three crosses at each age and treatment. Different lower case letters and asterisks represent significant statistical differences (p < 0.05).

A significant (p < 0.001) age × salinity interaction was found for the relative expression of the thyroid hormone receptor *thrαA* (Fig 4A), while *thrαB* and *thrβB* were not significantly affected by salinity (Fig 4C and 4E). For the latter two genes, age was the main factor influencing gene expression. Expression levels of *thrαB* increased throughout ontogeny (Fig 4B), while expression of *thrβB* increased from 2 to 4 dph and remained at steady levels beyond that (Fig 4D; both p < 0.0001). Moreover, larval age and salinity influenced genes involved in deiodination (*dio1-3*). Here, expression of *dio1* and *dio2* increased throughout ontogeny (Fig 4F and 4H), while expression of *dio3* decreased from 2 to 6 dph and increased again beyond that until 12 dph (Fig 4J; all p < 0.0001). However, *dio3* was the only gene in this functional group that was influenced by salinity, as its expression was reduced when salinity was decreased on 3 dph and at 2 psu/day (p < 0.02; Fig 4K).

#### Energy metabolism

Energy metabolism related genes (*atp6*, *cox1*) were found to be steadily expressed within the endogenous feeding period (2 to 12 dph) and at all salinities, with no significant differences occurring among treatments (Fig 4L–4O).

## Discussion

European eel is critically endangered with diminishing glass eel recruitment [2]. Thus, there is an urgent need to artificially produce offspring and improve survival during ELH of this species. Latest efforts have focused on extrinsic (environmental) factors such as light [14] and temperature [13, 45], while only eggs and embryos have been assessed regarding salinity effects [36]. In the closely related Japanese eel, a major improvement in larval captive rearing has been attributed to reduced salinity regimes [15, 46]. Therefore, this study investigated the effect of salinity on artificially produced European eel larvae (from a morphological and molecular point of view), to elucidate functionality and timing of osmoregulation related processes affecting ELH, in order to ultimately determine optimal conditions for rearing European eel pre-leptocephalus larvae in aquaculture. To accomplish this, we reared offspring throughout the endogenous feeding window (0–12 dph) and compared larvae reared at 36 psu (control), which is close to the salinity occurring in the Sargasso Sea [19], with larvae reared under reduced salinity conditions. Here, salinity was decreased on 0 or 3 dph and at rates of 1, 2 or 4 psu/day, resulting in six different salinity treatments (01, 02, 04, 31, 32 and 34).

### Mortality and biometry

Generally, we observed that reducing salinity, especially on 0 dph at 4 psu (fastest reduction) resulted in reduced mortality, increased body area, higher growth, reduced oil drop utilization and improved growth efficiency of the European eel larvae. Similar observations have been reported for Japanese eel, where better larval growth and survival were reported when reared in 50% reduced salinity [15]. Thereafter, it was shown that reducing seawater salinity (with an osmolality of ~1050 mOsm kg^-1^ H_2_O) to ~50%, facilitated an iso-osmotic environment for eel larvae with a tissue osmolality of 360 to 540 mOsm kg^-1^ H_2_O [23]. Concurrently, it has been argued that reducing salinity probably increased energy availability due to lower osmoregulatory expenses, enabling the survival of weaker larvae, which would not survive in a high salinity environment [46]. Additionally, [47] reported increased spinal deformities at a high salinity level (42 psu). In our study, we observed the highest incidence of spinal curvature (> 50%) and emaciation (> 80%) when larvae were reared at 36 psu (control) compared to < 5% and < 10% respectively, when salinity was reduced on 0 dph and at 4 psu/day (treatment 04). However, it has also been reported that rearing eel larvae at reduced salinity (< 33 psu), causes other deformities, such as pericardial edema and abnormal lower jaw [47]. The results of our study are in agreement with those findings, as we also observed an increased number of larvae with pericardial edema in the salinity reduced treatments. Thus, we demonstrate that reducing salinity was a tradeoff process, which improved survival and growth but also induced an edematous state of the larval heart. Hence, we consider that reducing salinity on 3 dph and at 2 psu/day towards iso-osmotic conditions, results in a more balanced mortality/deformity ratio. However, the persistency of the edematous state of the larval heart and if or how it represents an obstacle in further larval development needs to be clarified *via* future investigations.

### Thyroid metabolism

In order to evaluate the underlying molecular backgrounds relating to the observed biometric changes, we targeted genes involved in the thyroid metabolism, which is directly linked to growth and development of fish [48]. The importance of the thyroid endocrine system, especially during metamorphosis, has been documented in the Japanese conger eel, *Conger myriaster* [49], while thyroid hormone treatment is applied to coordinate a synchronized metamorphosis in Japanese eels [50]. Moreover, it was shown that the thyroid hormone pathway is involved in the early life development of Japanese [51] as well as European eels and that genes encoding thyroid hormone receptors and deiodinases show sensitivity to extrinsic (environmental) stimuli, such as temperature [45]. In the current study we observed that an early and fast reduction of salinity from 0 dph onwards increased *thrαA* levels, which is an underlying molecular response that can potentially be correlated to growth efficiency, which was also highest in this treatment. Moreover, the expression of *dio3* was also influenced by the salinity change, which would be in accordance with previous observations, where the outer ring deiodination showed sensitivity to salinity in rainbow trout, *Oncorhynchus mykiss* [52]. Furthermore, all genes relating to thyroid metabolism, investigated in this study, were affected by larval age and showed differentially expressed patterns throughout ontogeny, which are in accordance with previous reported results in European eel larvae [45] and thus further support the involvement of the thyroid endocrine system during ELH of this species.

### Osmoregulation

To further support our findings, we followed the relative expression of genes involved in ion transport. Here, we targeted the NKCC subfamily of the chloride cation cotransporter gene family, which mediates the electroneutral cotransport of Na^+^, K^+^ and Cl^-^ ions and is known to be involved in ion absorption and/or secretion as well as in cell volume homeostasis [27]. A secretory (*nkcc1*) and an absorptive (*nkcc2*) subtype (with isoforms for each) have been previously identified in adult European eel [53]. Of these, the *nkcc1α* isoform showed a wide range of tissue distribution, whereas *nkcc1β* was predominantly expressed in the brain [54]. Moreover, the *nkcc2α* isoform was rather restricted to renal tissues, whereas the *nkcc2β* isoform predominated in the intestine and urinary bladder [55]. In our study, we observed that *nkcc1α* and *nkcc2β* expression decreased with salinity reduction, which indicates a downregulation of the active Na^+^, K^+^, and Cl^-^ transport. As this mechanism requires energy, a lowered transcellular ion transport can probably lead to reduced cellular energy consumption. Furthermore, the ionoregulatory ability of the larvae seemed to increase throughout ontogeny (i.e. increased expression of *nkcc2α* and *nkcc2β*), which is probably coupled to the increasing functionality of the associated tissue (kidney and gut respectively) during organogenesis.

Ionoregulation is tightly coupled to water flow across membranes, where aquaporins form pores to selectively facilitate rapid transport and exchange of water molecules [28]. In eurohaline fish, such as the sea-bass (*Dicentrarchus labrax*), aquaporins facilitate the water uptake in the intestinal tract and the reabsorption of water in the kidney to counteract dehydration in response to high salinity [56]. Similarly, in relation to seawater acclimation, branchial *aqp3* was downregulated, but intestinal *aqp3* was unchanged [57], while renal *aqp1*, *aqp1dup* and *aqpe* were downregulated [58], but intestinal *aqp1* and *aqpe* were upregulated [59] in European eel. Probably the high osmotic water loading through the gills and the associated excretion of large volumes of dilute urine (needed in freshwater) are no longer necessary in a saline environment (gill and renal expression downregulation), while on the contrary, the increased ingestion of seawater and the accompanied transcellular movement of large quantities of salts in the gastro-intestinal track, require concomitant changes in transcellular water transport (intestinal expression upregulation) [60]. In our study we observed a down-regulation of *aqp1dup* and the two aquaglyceroporins (*aqp3*, *aqpe*) when salinity was reduced early and fast (from 0 dph and at 4 psu/day), which is an indication that larvae acclimated their hydromineral regulation in response to lower salinity by reducing water retention. However, as gills are not present and the kidney as well as the intestine are undeveloped and only gain functionality during this developmental period, the tissue specific functionality of this molecular mechanism in relation to salinity change and the potential sensitivity shift among developmental stages remain to be clarified.

### Stress response

In order to analyze the cellular stress response in European eel larvae we targeted *hsp70* and *hsp90*, which are expressed (in response to stress) to counteract physiological injury and reduce trauma [13, 29]. The *hsp* function, is commonly associated but not restricted to heat stress and has been recognized to have a more universal role in response to a number of stressors [29]. As such, *hsps* have been shown to be sensitive to changes in salinity, where lowest levels of *hsps* were found in Black sea bream (*Mylio macrocephalus*) reared in an iso-osmotic salinity and highest levels when reared bellow (hyper-osmotic) or above (hypo-osmotic) the osmotic homeostasis [61]. A similar response was observed in our current study, where larval *hsp90* levels decreased in the treatments were salinity was reduced in 2 or 4 psu/day compared to larvae reared in full strength seawater (36 psu). This suggests that eel larvae reared in iso-osmotic conditions are less stressed probably due to lower energetic costs in maintaining cellular homeostasis.

### Energy metabolism

Thereafter, we evaluated energy levels in European eel larvae in response to environmental salinity changes by targeting genes involved in the OXPHOS pathway and associated to energy metabolism in teleost fishes [31]. Here, the expression levels of ATP-synthase and cytochrome-c-oxidase were stable throughout the entire developmental period investigated and independent of salinity levels, suggesting that energy production was stable across all salinity treatments. As such, in the light of decreased osmotic demands and stress (i.e. lower *hsp*, *nkcc*, and *aqp* expression), European eel pre-leptocephalus larvae reared at iso-osmotic salinity conditions seem to have a higher energy availability compared to larvae reared in full strength seawater (36 psu), which can then be utilized more efficiently to increase growth and survival.

### Conclusion

To summarize, this study morphologically and molecularly elucidated osmoregulation related processes and together with the consideration of the most balanced mortality/deformity ratio, we conclude that salinity reduction benefits European eel larvae in terms of lower mortality and improved growth efficiency, which is likely facilitated by an energy surplus associated to lower osmoregulation demands. Hence, the overall knowledge gained from this study adds to our understanding of underlying biological mechanisms during ELH of European eel and provides a promising step towards optimized rearing conditions for European eel pre-leptocephalus larvae as well as in the strive for sustainable aquaculture of this species. Nevertheless, as the studied molecular parameters foreshadow an adequate subsequent larval development, likely due to a global lower osmoregulatory cost, further research is needed in order to verify the energetic developmental costs in contrasted salinity scenarios and evaluate the long-term morphological, molecular and physiological effects.

## Supporting information

S1 File Data. (XLSX)
